# Identification of Genes Involved in Resistance to Flavescence Dorée Disease of Grapevine: A Model Study Using *Arabidopsis thaliana*

**DOI:** 10.1111/mpp.70273

**Published:** 2026-05-18

**Authors:** Marika Rossi, Luciana Galetto, Simona Abbà, Nicola Bodino, Cristina Marzachì, Sabrina Palmano

**Affiliations:** ^1^ Institute for Sustainable Plant Protection – National Research Council of Italy IPSP‐CNR Torino Italy; ^2^ Department of Agriculture, Forest and Food Sciences (DiSAFA) University of Turin Turin Italy

**Keywords:** differential gene expression, electrical penetration graph EPG, phytoplasma, reverse genetics, RNAseq, susceptibility

## Abstract

Flavescence dorée (FD), caused by FD phytoplasma (FDp) and transmitted by the leafhopper *Scaphoideus titanus*, is a quarantine disease that seriously threatens viticulture across Europe. Research on resistance or tolerance to FDp in grapevine is limited by the perennial nature of the host, high cultivar variability and the univoltine life cycle of the insect vector. To overcome these constraints, we employed *Arabidopsis thaliana* as a model host to identify genes conferring resistance to FDp. RNA sequencing (RNA‐seq) was first used to identify genes deregulated in *A. thaliana* during infection by either the woody host pathogen FDp or the herbaceous host pathogen Chrysanthemum yellows phytoplasma (CYp). A subset of these genes was then validated by monitoring their expression in the FDp– *A. thaliana* pathosystem at various time points during infection. Five genes were consistently deregulated upon infection, and functional analysis of the corresponding mutants revealed two lines with significantly reduced susceptibility to FDp compared with the wild type. The double mutant combining these two genes exhibited a similar phenotype, suggesting functional convergence. Electrical penetration graph (EPG) analysis indicated that this resistance is independent of vector feeding behaviour, pointing to a genetically determined defence mechanism rather than antixenosis. These findings uncover novel components of plant defence against phytoplasma infection and establish *A. thaliana* as a valuable system for dissecting the molecular basis of tolerance to FDp. We discussed how the genes we identified represent promising targets for developing sustainable, phytoplasma‐resistant grapevine varieties.

## Graphical abstract

*Arabidopsis thaliana* genes conferring resistance to flavescence dorée (FD) phytoplasma were identified, revealing vector‐independent defence mechanisms and providing potential targets for breeding FD‐resistant grapevine.

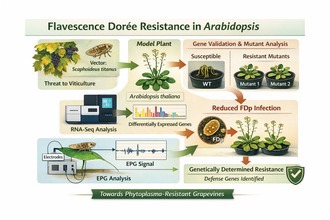

## 1 Introduction

Flavescence dorée (FD) is a quarantine disease of grapevines that poses an economic threat to the wine production industry (European Food Safety Authority (EFSA) et al. 2025). Since it was first reported in France in the 1950s (Caudwell 1990), FD has spread widely throughout most European viticultural regions and continues to extend its reach to new geographical areas due to climate change and global warming (Brooks et al. 2022).

FD is caused by a phytoplasma (FDp), which is a wall‐less bacterium belonging to the Mollicutes class. It is transmitted to grapevines by various phloem‐feeding hemipterans, the most relevant of which is the leafhopper *Scaphoideus titanus* (Gonella et al. 2024). The control of FD relies on compulsory insecticide treatments to reduce the vector population, which has a severe impact on human and environmental health. Furthermore, the elimination of infected plants and their replacement with certified plant material significantly increases management costs, offering only temporary relief from the disease, as new plants can become infected quickly, particularly in areas with a high infection pressure.

For these overall reasons, alternative FD management strategies are needed and various strategies are being studied to improve the sustainability of vineyards (Oliveira et al. 2019). Among the most promising approaches are studies focusing on the genetic resources of *Vitis vinifera* (Mian et al. 2023; Paineau et al. 2025). Indeed, the exploitation of genetic resistance in grapevine has been reported for various diseases caused by different pathogens, primarily fungi (Armijo et al. 2016; Merdinoglu et al. 2018; Capriotti et al. 2020; Sosa‐Zuniga et al. 2022). No genetic resistance to FD has yet been described, although cultivars with different degrees of susceptibility have been identified (Eveillard et al. 2016; Ripamonti et al. 2021). Therefore, investigating how plants defend themselves against phytoplasma infection could reveal molecular traits associated with resistance or tolerance to FDp. Studies on the interaction between FDp and grapevine on their natural hosts can be challenging due to the woody host's perennial life cycle, the genomic and phenotypic features of different grapevine cultivars, and the monovoltine life cycle of the main insect vector. In this study, we used the model plant *Arabidopsis thaliana* to investigate the plant's response to phytoplasma infection. Due to its small size and short generation time, *A. thaliana* enables rapid genetic studies; moreover, its genome has been sequenced, and numerous mutants with altered phenotypes and biochemistry have been mapped and characterised (Van Norman and Benfey 2009).

*Arabidopsis thaliana* has previously been used as a model plant to study interactions between plants and phytoplasmas, primarily in the context of the ‘*Candidatus* Phytoplasma asteris’ pathogen system (MacLean et al. 2011; Sugio, Kingdom, et al. 2011; Sugio, MacLean, et al. 2011; Pacifico et al. 2015; Pagliari et al. 2017; Bernardini et al. 2020, 2022). However, no research has been conducted on FDp.

In the present study, we used RNA‐seq to identify regulated genes in *A. thaliana* infected by a strain of ‘*Ca*. P. asteris’ or FDp in single infections. The sets of concordantly and discordantly regulated genes were integrated with the available literature to identify conserved genes in *A. thaliana* that are regulated following infection by pathogens other than phytoplasmas. The altered expression of the selected genes was confirmed and monitored in the early stages and over time FDp infection in *A. thaliana* Col‐0 under controlled conditions. Finally, we validated the biological role of the confirmed deregulated genes using a reverse genetics approach to phenotype the corresponding *A. thaliana* mutants for FDp susceptibility. We also evaluated the vector feeding behaviour in the least susceptible mutant to rule out the antixenotic activity of the plant against insects.

## 2 Results

### 2.1 Gene Expression in FDp‐Infected *A. thaliana*

**FIGURE 1:**
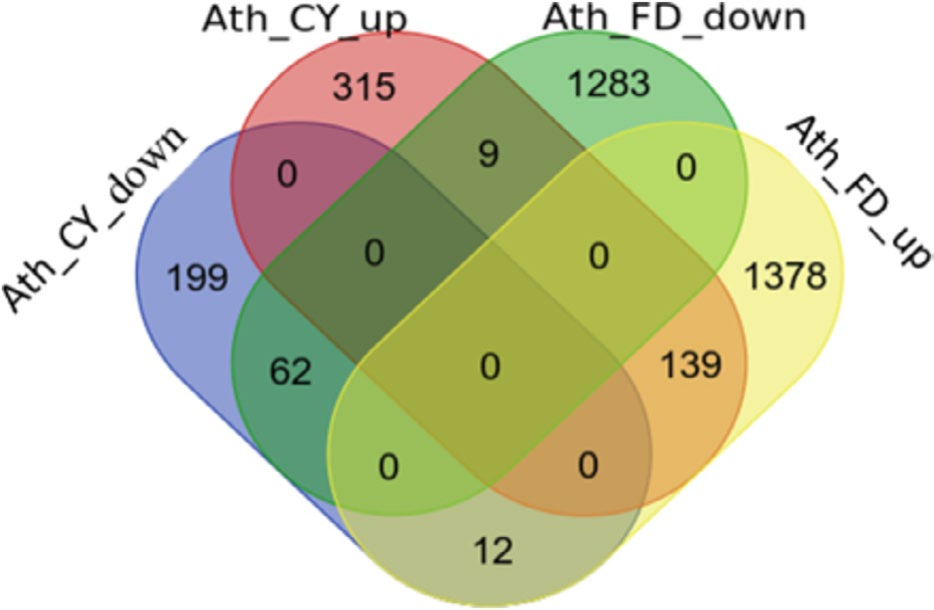
Venn diagram obtained by comparing next‐generation sequencing (NGS) data from *Arabidopsis thaliana* infected with chrysanthemum yellows phytoplasma (CYp) or flavescence dorée phytoplasma (FDp), in relation to the healthy plant, showing the number of concordantly and discordantly regulated genes.

RNA‐seq data from *A. thaliana* plants infected separately with ‘*Ca*. Phytoplasma asteris’ isolate Chrysanthemum yellows phytoplasma (CYp; Pacifico et al. 2015) or FDp were used to identify genes deregulated upon phytoplasma infection and to monitor their expression dynamics at early and subsequent time points in new FDp infection experiments. The subset of genes selected for expression analysis derived from lists of genes differentially expressed during CYp or FDp infection (Table S1). Comparison of these datasets enabled the identification of genes displaying concordant regulation (similarly up‐ or downregulated in response to both phytoplasmas) and discordant regulation (upregulated in response to one phytoplasma but downregulated in response to the other) (Figure 1).

Additionally, one and five genes uniquely regulated under CYp or FDp infection, respectively, were included to discriminate general defence responses from pathogen strain‐specific responses. Gene selection also considered literature evidence supporting their role in plant defence, and three additional *A. thaliana* genes associated with responses to biotic stress factors other than phytoplasmas were included. We further assessed whether any of the selected genes were reported in the grapevine/FDp pathosystem (Pagliarani et al. 2020; Casarin et al. 2023; Corio‐Costet et al. 2025). Finally, expression patterns of 13 genes belonging to the four metabolic pathways ‘Cell development’, ‘Hormone metabolism’, ‘Plant growth regulation’ and ‘Plant defence’ (Table 1) were evaluated in leaves of *A. thaliana* plants exposed to *Euscelidius variegatus* individuals infected with FDp (FD), uninfected insects (K), or not exposed to insects (H).

**TABLE 1:** List of the *Arabidopsis thaliana* genes selected for gene expression analyses upon flavescence dorée phytoplasma (FDp) infection.

Function	Gene name	Gene accession (TAIR codes)	Description (TAIR)	Preliminary bioinformatic analysis		Reference (pathogen/host plant)
				CYp	FDp	
Plant stress and defence	*NHL26* (*LEA*)	AT5G53730	Phloem specific membrane protein. Overexpression causes defects in sugar export with reduced levels of phloem sugars	NA	Up	FDp/*A.thaliana*; this study FDp/*V. vinifera*; Pagliarani et al. (2020)
	*ATL2*	AT3G16720	Encodes a RING‐H2 zinc finger protein that functions as an E3 ubiquitin ligase	NA	NA	*Alternaria brassicicola/A.thaliana*; Kim et al. (2024) *Plasmopara viticola/V. vinifera*; Vandelle et al. (2021)
	*BPI*	AT1G04970	Encodes one of the two LBP/BPI related proteins that biNA to LPS directly and regulate PR1 expression. Putative BPI/LBP family protein	NA	Up	FDp/*A. thaliana*; this study
	*PAD4*	AT3G52430	Encodes a lipase‐like gene that is important for salicylic acid signalling and function in resistance (R) gene‐mediated and basal plant disease resistance	NA	NA	Downy mildew/*A.thaliana*; Glazebrook et al. (1997) *Pseudomonas syringae* pv. *maculicola*/*A. thaliana*; Zhou et al. (1998)
Hormone metabolism	*JAZ7*	AT2G34600	Encodes JAZ7, a key regulator in alternative splicing in the jasmonate signalling pathway, alone and in collaboration with other regulators	Down	NA	CYp/*A. thaliana*; this study FDp/*V. vinifera*; Pagliarani et al. (2020)
	*CKX5*	AT1G75450	Encodes CKX5, a protein whose sequence is similar to cytokinin oxidase/dehydrogenase, which catalyses the degradation of cytokinins.	Down	Up	CYp/*A. thaliana*; this study FDp/*A. thaliana*; this study FDp/*V. vinifera*; Pagliarani et al. (2020)
Plant growth regulation	*AIG2‐like*	AT3G02910	AIG2‐like (avirulence induced gene) family protein	NA	Up	FDp/*A. thaliana*; this study
	*LHT1*	AT5G40780	Encodes LHT1 (lysine histidine transporter), a high‐affinity transporter for cellular amino acid uptake in both root epidermis aNA leaf mesophyll	NA	Up	FDp/*A. thaliana*; this study FDp/*V. vinifera*; Pagliarani et al. (2020)
	*MFSP*	AT2G16660	Encodes Major facilitator superfamily protein	Down	Up	CYp/*A. thaliana*; this study FDp/*A. thaliana*; this study
	*PLE*	AT5G51600	Encodes a 65‐kDa microtubule‐associated protein 3 with a role in organizing the mitotic microtubule array during both early and late mitosis in all plant organs	Up	Up	CYp/*A. thaliana*; this study FDp/*A. thaliana*; this study FDp/*V. vinifera*; Pagliarani et al. (2020)
Cell development	*GSL5*	AT4G03550	Encodes a callose synthase that is required for wouNA aNA papillary callose formation in response to fungal pathogens *Erysiphe* and *Blumeria*. Contributes to PAMP‐induced basal defence	NA	NA	*Hyaloperonospora parasitica*/*A. thaliana*; Nishimura et al. (2003) CYp/*A. thaliana*; Pagliari et al. (2017)
	*PAP1* (*FIB*)	AT4G04020	Encodes the Fibrillin precursor protein. Regulated by abscisic acid response regulators. Involved in abscisic acid‐mediated photoprotection	NA	Up	FDp/*A. thaliana*; this study FDp/*V. vinifera*; Pagliarani et al. (2020)
	*LTP*	AT3G53980	Predicted to encode a PR (pathogenesis‐related) protein. Belongs to the lipid transfer protein (PR‐14) family	Up	Up	FDp/*A. thaliana*; this study CYp/*A. thaliana*; this study FDp/*V. vinifera*; Pagliarani et al. (2020)

*Note:* Most of the genes were selected as being differentially regulated (Up/Down) during either flavescence dorée phytoplasma (FDp) or ‘*Candidatus* Phytoplasma asteris’ Chrysanthemum yellows isolate (CYp) infection compared to healthy controls. Three genes, not regulated during phytoplasma infection in *A. thaliana* (NA/NA), were selected based on existing literature as differentially regulated during biotic stress in *A. thaliana* and potentially in *Vitis vinifera*.

**FIGURE 2:**
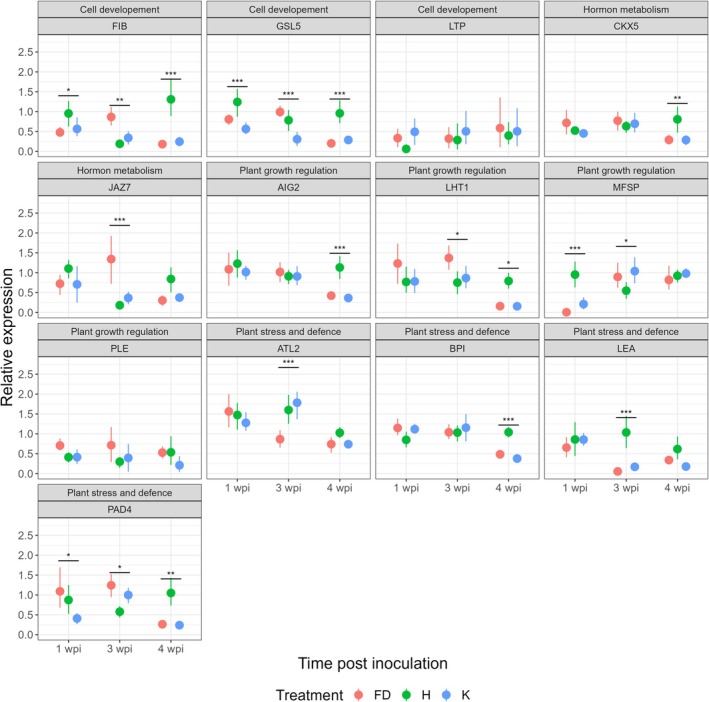
Relative expression in *Arabidopsis thaliana* Col‐0 of the selected genes for each of the four categories resulting from the preliminary bioinformatic analyses. Three treatments were applied: plants exposed to flavescence dorée phytoplasma‐infected (FD, red) or healthy (K, blue) *Euscelidius variegatus* , and not exposed to insects (H, green). Total RNA was extracted at 1, 3 and 4 weeks post‐infection (wpi) from leaves and analysed by reverse transcription‐quantitative PCR using the primers described in Table S2. For each gene and sampling time combination, asterisks show statistically significant differences between treatments (Gaussian GLM: *p < 0.05, **p < 0.01, ***p < 0.001). Error bars represent standard error of the mean expression value for each treatment.

Because the RNA‐seq analysis was conducted at 10 weeks post‐inoculation, it captured gene expression at a single and advanced stage of infection. To better understand the temporal dynamics of gene regulation, selected genes were therefore analysed at earlier stages of infection. Samples were collected at three time points: 1‐, 3‐ and 4‐weeks post‐insect exposure (wpi; Figure 2, Table S2). As the selection of appropriate reference genes is critical for reverse transcription‐quantitative PCR (RT‐qPCR) data normalisation, we evaluated the expression stability of five potential reference genes: *SYP43*, *TOPP4*, *TIP‐41like* and *SAND family* selected according to literature analyses (Table S3), and *DNAJ* according to our RNA‐seq data on *A. thaliana* infected by CYp or FDp. According to the amplification efficiency of each primer pair (Table S3) and geNorm output values (0.74, 1.02, 0.81, respectively), *SYP43*, *TIP‐41like* and *DNAJ* were selected as the most stable reference genes in our experimental conditions.

**FIGURE 3:**
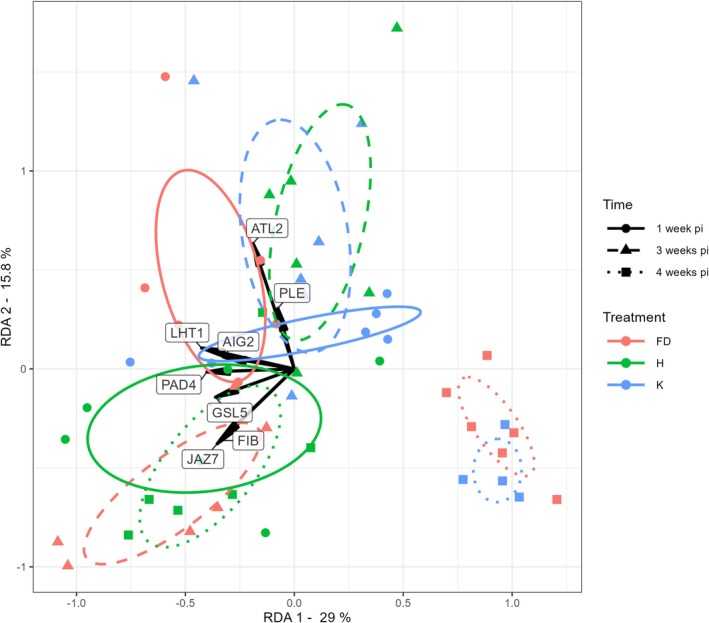
Multivariate redundancy analysis (RDA) of the relative expression of the selected genes in leaves of *Arabidopsis thaliana* exposed to flavescence dorée phytoplasma‐infected (FD, red) or healthy (K, blue) *Euscelidius variegatus*, and not exposed (H, green) to insects at different weeks post‐inoculation (wpi). Similarity index: Euclidean distance; association method: Ward. Clusters were grouped with standard deviation ellipses bordered by lines with different hatches according to the sampling data (full line: 1 wpi, dashed line: 2 wpi, dotted line: 4 wpi).

We explored the variability in relative gene expression for the selected genes following the three treatments by univariate (ANOVA) (Figure 2, Table S4) and multivariate (redundancy analysis, RDA) (Figure 3, Table S5) statistics. RDA showed that treatment, time after inoculation, and their interaction significantly contributed to the observed variation in gene expression profiles. Plants exposed to healthy insects (K) often displayed distinct expression patterns compared to unexposed controls (H), particularly at 1 wpi for *GSL5* and *MFSP*, and at 4 wpi for *FIB*, *GSL5*, *PAD4*, *AIG2*, *BPI* and *CKX5* (Figure 2, Table S5), indicating a generic transcriptional response to leafhopper feeding. These results showed that comparisons between plants exposed to infected insects (FD, red in Figures 2 and 3) and those exposed to healthy insects (K, blue in Figures 2 and 3) would better identify genes specifically responding to phytoplasma infection in *A. thaliana* .

According to both ANOVA and RDA of the relative gene expressions (Figures 2 and 3; Tables S4 and S5), two genes belonging to the ‘cell development’ category, *FIB* and *GSL5*, were upregulated in FD compared to K treatment at 3 wpi; *JAZ7*, belonging to ‘Hormone metabolism’, was also upregulated in FD compared to K at 3 wpi; two more genes belonging to ‘Plant stress and defence metabolism’ were downregulated at 3 wpi (*ATL2*) and upregulated (*PAD4*) at 1 wpi. These results indicated that the most relevant interval to measure differences in relative gene expression between FD and K was at 3 wpi, when the infection was well established and the plants were not yet undergoing senescence effects.

### 2.2 Susceptibility of Selected *A. thaliana* Mutants to FDp

According to the differential expression analysis reported above, *Fib, Gsl5, Jaz7, Atl2* and *Pad4* mutants were selected to explore the genotype susceptibility to FDp infection. *Ltp, Ckx5* and *Msfp* mutants were added to the experiments as controls, as *LTP* (‘Cell development’ category) gene expression was constant irrespective of treatment and sampling date, while *CKX5* (‘Hormone metabolism’) and *MSFP* (‘Plant growth regulation’) genes were differentially expressed at least at one sampling date only in H treatments (Table S4). *Atl2* mutant seeds never germinated, and this genotype was not further explored (Table S6).

Following a first inoculation trial with infective *E. variegatus* , the genotypes showing similar FDp infection rates as the wild type were not further tested, while those showing different susceptibilities compared to the control (*Jaz7*, *Gsl5* and *Pad4*) were subjected to a second inoculation trial to confirm the results. Similarly, we tested the susceptibility of the double mutant *Pad4/Pmr4* (Nishimura et al. 2003), which carries the two most promising mutations identified in our study: *Pad4* and *Gsl5* (Table 2; Table S7).

**TABLE 2:** Number of inoculated and flavescence dorée phytoplasma (FDp)‐infected plants (wild type and mutants).

Genotype	Number of inoculated plants	Number of FDp‐positive plants	Mean FDp GU/ng ± SEM
Col‐0 (wild type)	134	70	4.84e+02 ± 1.69e+02
*Jaz7*	63	45	5.39e+03 ± 4.56e+03
*Pad4*	56	17	1.60e+02 ± 4.87e+01
*Gsl5*	61	20	1.36e+02 ± 6.09e+01
*Pad4/Pmr4*	61	11	1.12e+02 ± 4.54e+01

*Note:* Phytoplasma loads are expressed as FDp genome units (GU) per ng of total plant DNA, together with the standard error of the mean (SEM).

The FDp titre in the infected plants ranged from 1.54e+00 to 2.05e+05 GU/ng plant DNA (Table 2), without significant differences among genotypes.

**FIGURE 4:**
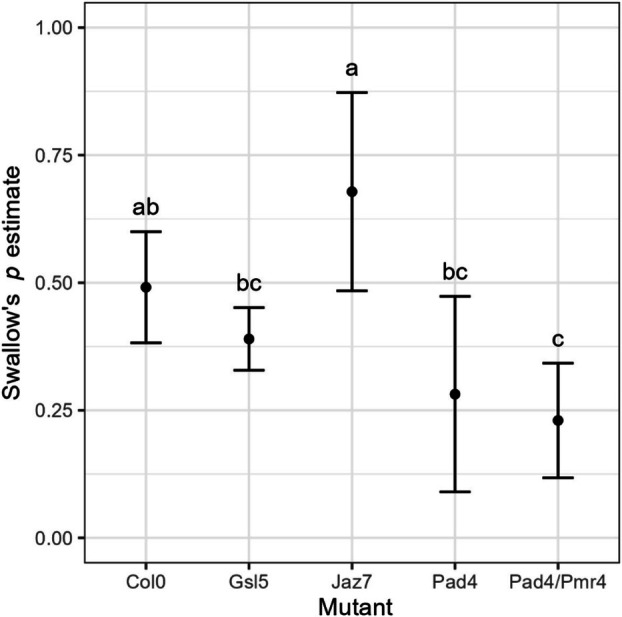
Mean (±CI) probability (Swallow's *p*) of a single infected insect to successfully inoculate flavescence dorée phytoplasma to each *Arabidopsis thaliana* genotype during a 7‐day inoculation access period (IAP). Mean infection rates of mutants sharing a letter above CI bars did not differ significantly at the significance level *α* = 0.05.

The normalisation of *A. thaliana* susceptibility was estimated as the probability (Swallow's *p*) of a single infected insect to successfully inoculate FDp to a recipient plant during the 7‐day inoculation access period (IAP), calculated by applying a modified Swallow's *p* formula (Swallow 1985). The estimated average probability of infection of *Jaz7* plants was higher, although not significantly, than Col‐0 plants (Swallow's *p* = 0.491 ± 0.055, CI: 0.382–0.6) and significantly higher (Swallow's *p* = 0.678 ± 0.099, CI: 0.484–0.873) than both *Gsl5* (Swallow's *p* = 0.390 ± 0.031, CI: 0.328–0.451) and *Pad4* (Swallow's *p* = 0.281 ± 0.098, CI: 0.09–0.473) mutants (Table S8). The double mutant *Pad4/Pmr4* showed a lower probability of becoming infected with respect to the control, in line with the two individual mutant lines (Swallow's *p* = 0.230 ± 0.057, CI: 0.1118–0.342) (Figure 4, Table S8).

### 2.3 Analysis of *E. variegatus* Feeding Behaviour on *A. thaliana* Mutants

**FIGURE 5:**
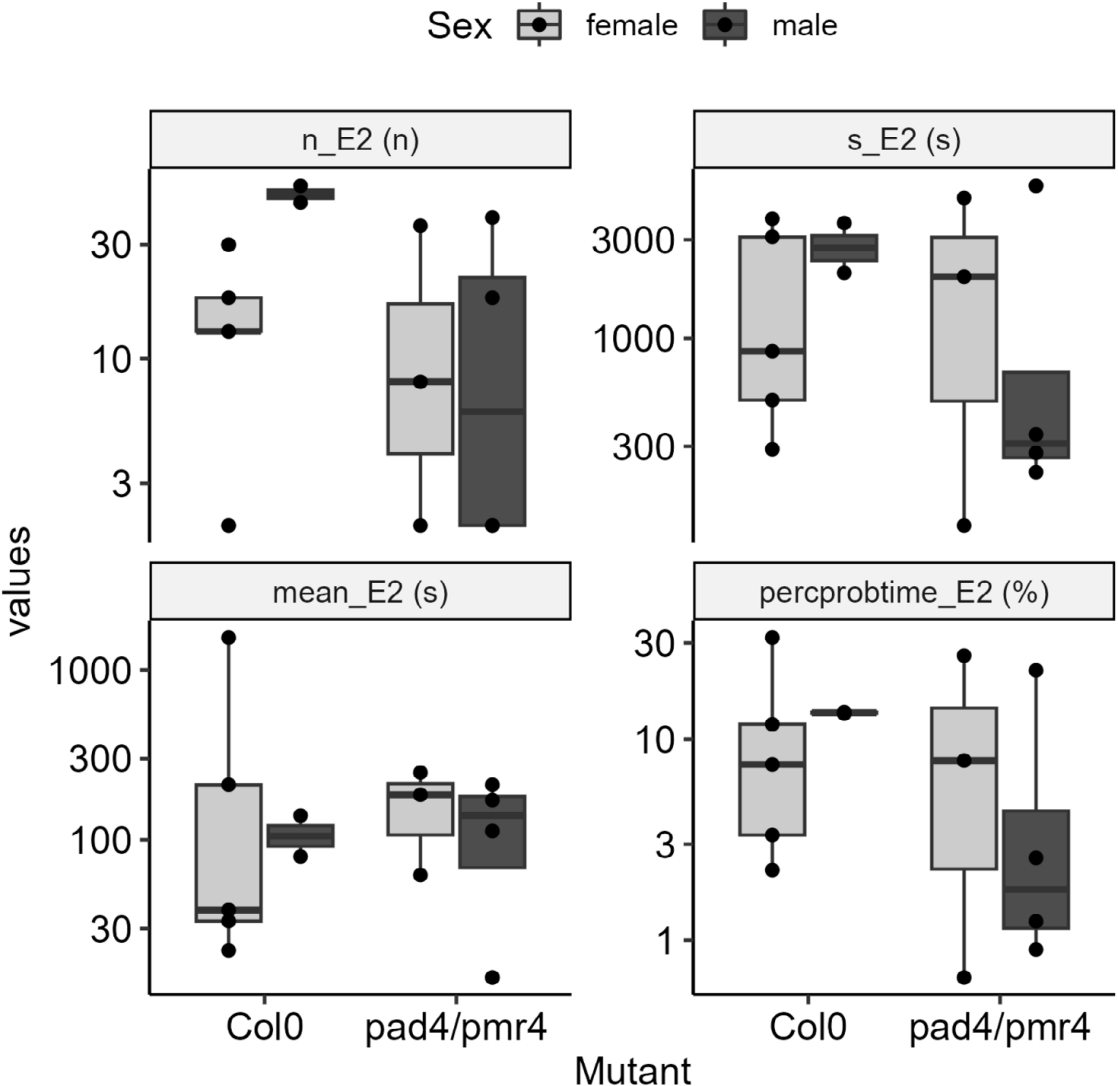
Variability in a selection of electrical penetration graph (EPG) variables related to recordings with phloem phases between *Arabidopsis thaliana* Col‐0 and mutants *Pad4/Pmr4*: n_E2: number of phloem ingestions; s_E2: total duration of phloem ingestions [s]; meanE2: mean duration of a single event of phloem ingestion (s); percprobtime_E2: percentage of probing time spent in phloem ingestion (%).

We tested by electrical penetration graph (EPG) the feeding behaviours of the lab vector of FDp, *E. variegatus* , on *A. thaliana* Col‐0 (control) and *Pad4/Pmr4* (mutant) plants to explore whether altered nutrition of the vector might explain differences in the FDp susceptibility of the two genotypes (Table S9). During the probing phase (stylet inserted into the leaf tissue), insects spend most of the recording time (usually 8 h = 28,800 s) (median ± SE = 1.37e+04 ± 3.21e+03 s; Table S9) in a stage characterised by tissue exploration (pathway phase) rather than active feeding (phloem phase E2). Such prolonged non‐probing and pathway phases clearly indicate a low palatability of *A. thaliana* for the insect. The proportion of recordings exhibiting phloem phases were similar among tested mutants (Col‐0: 22.6%, 7 out of 31; *Pad4/Pmr4*: 21.9%, 7 out of 32), and no significant differences in feeding behaviour parameters were observed among mutants, sex of insects or their interaction (Figure 5, Table S10). Number and duration of phloem feeding events during the recording (i.e., 8 h) were on average 24.9 ± 7.0 (interquartile range, IQR: 13–37.5) and 296 ± 210 s (IQR: 35.9–175.0 s), respectively, on Col‐0 plants and 15.3 ± 6.1 (IQR: 2–27) and 144.0 ± 31.6 (IQR: 87.3–197) on *Pad4/Pmr4* mutants. Graphical summaries of temporal progression of behavioural phases show that the percentage of leafhoppers in phloem phase was quite low (Col‐0: 12.0% ± 3.75%, IQR 5.41%–13.5%; *Pad4/Pmr4*: 8.73% ± 4.07%, IQR 1.07%–14.9%) during the entire duration of the recording, without a significant difference between *A. thaliana* genotypes (not shown). No statistically significant differences were highlighted for the parameters related to phloem nutrition. The results highlighted that *A. thaliana* is a suboptimal plant host for *E. variegatus* . Indeed, insects spent on average 30% of their time not feeding (non‐probing phase), with their stylets not inserted into the leaf tissue.

## 3 Discussion

In this study, we demonstrated that two distinct phytoplasmas infecting *A. thaliana* trigger complex patterns of gene expression changes, as revealed by RNA‐seq analysis. A set of genes differentially regulated in a pathogen‐specific manner or affected as part of a general plant defence response to biotic stress, plus three *A. thaliana* genes known to be involved in responses to other biotic stress factors, were included in subsequent gene expression studies. In detail, *ATL2* was selected for further analysis based on its role in activating systemic acquired resistance (SAR) when constitutively expressed in *A. thaliana* (Serrano and Guzmán 2004) and its association with downy mildew resistance in grapevine (Vandelle et al. 2021). *PAD4* and *GSL5* were selected due to their involvement in responding to various pathogens, including fungi and bacteria (Glazebrook et al. 1997; Zhou et al. 1998; Nishimura et al. 2003). The regulation of the selected genes was validated at three stages of *A. thaliana* early infection with FDp, and five of these were differentially expressed compared to the control group, consisting of plants exposed to healthy vectors. This approach enabled exclusion from further analyses of genes deregulated only in the presence of leafhoppers feeding on the plant. As a result of this study, we phenotyped specific *A. thaliana* mutants for FDp resistance to confirm the involvement of the regulated five genes in the plant's response to the phytoplasma.

Time‐course gene expression analysis showed that FDp infection elicits stage‐specific transcriptional regulation, allowing the distinction between genes consistently associated with infection and those related to transient or late‐stage responses. This approach also revealed significant deregulation in three key gene categories: cell development, hormone metabolism, and plant stress and defence.

### 3.1 Cell Development Genes

Among the ‘cell development’ gene category, *GSL5* and *FIB* showed coordinated upregulation at 3 wpi. The *GSL5* gene, also known as *PMR4* (POWDERY MILDEW RESISTANT4), encodes a callose synthase enzyme crucial for a plant's response to pathogens (Dong et al. 2008). In *A. thaliana* , this enzyme belongs to a family of 12 callose synthase genes (Verma and Hong 2001) and is upregulated in response to salicylic acid (SA), a key defence hormone (Vogel and Somerville 2000). Although callose deposition is a well‐known plant defence mechanism, its role against phytoplasmas appears complex. For example, the phloem‐specific callose synthase gene *Cal7* is deregulated in phytoplasma‐infected plants (De Marco et al. 2016), but an *AtCal7* knockout mutant shows increased susceptibility (Bernardini et al. 2022). Similarly, an uncharacterised callose synthase is upregulated in symptomatic canes of phytoplasma‐infected grapevines (Casarin et al. 2023). Interestingly, our findings reveal a different dynamic. The *Pmr4* mutant, which exhibits reduced callose deposition, demonstrated lower susceptibility to FDp than wild‐type plants. This aligns with previous observations that callose deposition is higher in the central veins of phytoplasma‐infected plants than in healthy ones and does not prevent the pathogen from spreading (Musetti 2009). Our result mirrors the *Pmr4* mutant's known resistance to the biotrophic fungus powdery mildew, where reduced callose production correlates with a stronger activation of SA‐ and other pathogen‐responsive genes (Glazebrook et al. 1997). This suggests that excessive callose deposition might not enhance defence responses against all pathogens but may hinder a more effective and coordinated response (Nishimura et al. 2003). Fibrillins (FIBs) enhance plant resilience to biotic and abiotic stresses (El‐Sappah et al. 2024) through accumulation of plastoglobules and triacylglycerols, which activate jasmonic acid (JA) biosynthesis in chloroplasts under stress conditions (Youssef et al. 2010). Despite this, the *Fib* mutant showed a similar FDp infection rate to the wild type. In the same metabolic category, deregulation of *LTP* during FDp infection of *A. thaliana* was not further confirmed in the time course gene expression study. *LTP* encodes a protein within the lipid transfer family, with a known role in plant defence (Gomès et al. 2003; Girault et al. 2008; Gao et al. 2022), but its expression is altered also during plant development (Edqvist et al. 2018).

### 3.2 Hormone Metabolism Genes

Within the ‘hormone metabolism’ gene category, only *JAZ7* was deregulated by FDp infection. The *JAZ7* gene encodes a JASMONATE ZIM‐domain (JAZ) protein, a negative regulator of the jasmonic acid (JA) signalling pathway, which is essential for plant defence against pathogens, environmental stresses and herbivorous insects (Browse and Wager 2012; Thatcher et al. 2016; Hanif et al. 2018). It also influences various physiological processes, including root growth and senescence (Hu et al. 2017; Howe et al. 2018). In stressed plants, activation of JA‐mediated responses occurs through degradation of JAZ proteins by the JA active form, JA‐Ile (Thireault et al. 2015; Han 2017). *A. thaliana* has 13 genes encoding JAZ proteins, and JAZ7 has a specific role in defence. Overexpression of *JAZ7* leads to increased susceptibility to pathogens such as *Pseudomonas syringae* and *Fusarium oxysporum* (Thatcher et al. 2016; Zhang et al. 2018). These results match our findings, as *Jaz7* mutant was significantly more susceptible to FDp than Col‐0 plants. While it may appear counterintuitive that a gene conferring increased susceptibility could be valuable for disease management, understanding the molecular mechanisms underlying host–pathogen interactions is crucial for developing effective control strategies. This is relevant given that the grapevine genome encodes 11 JAZ proteins regulated by various biotic stresses (Zhang et al. 2012). Indeed, a gene involved in the synthesis of JA (*VvLox2*) is upregulated in the FD‐poorly susceptible Merlot cultivar compared to the highly susceptible Cabernet Sauvignon (Corio‐Costet et al. 2025). Moreover, the heterologous expression of grapevine *JAZ7* in *A. thaliana* increases the resistance of the mutant to powdery mildew but not to *Botrytis cinerea*, supporting the role of this gene for future genetic approaches in grapevines (Hanif et al. 2018). Within the same metabolic category, the differential regulation of the CYTOKININ OXIDASE/DEHYDROGENASE 5 (*Ckx5*) gene in Ath‐CY and Ath‐FD systems was not confirmed in the subsequent gene expression study, although the gene is strongly activated upon infection with *B. cinerea* (Wang et al. 2024).

### 3.3 Plant Stress and Defence Genes

Within the ‘Plant stress and defense’ category, we investigated four genes, two of which, *PAD4* and *ATL2*, were deregulated by FDp infection at 3 and 1 wpi, respectively. Notably, the *Pad4* mutant exhibited reduced susceptibility to FDp compared to Col‐0. The PHYTOALEXIN DEFICIENT 4 (*PAD4*) gene encodes a lipase‐like protein that plays a central role in SA accumulation. PAD4 forms a functional heterodimer with Enhanced Disease Susceptibility 1 (EDS1), a critical regulator promoting basal and effector‐triggered immunity (Zhou et al. 1998; Pruitt et al. 2021). Host–pathogen interactions can modify plant development through pathogen effectors or host resistance responses. Phytoplasma effectors can actively reprogramme host development (Hogenhout and Loria 2008; Hogenhout et al. 2008; MacLean et al. 2011; Sugio, MacLean, et al. 2011; Sugio, Kingdom, et al. 2011; Furch et al. 2021; Omenge et al. 2021). Mutations in *PAD4* reduce resistance to bacterial pathogens such as *P. syringae* pv. *maculicola* ES4326 (Glazebrook et al. 1997). In contrast, *Pad4* mutants in this study exhibited reduced susceptibility to FDp compared to Col‐0. This response may result from alternative signalling pathways sustaining SA‐mediated defences. The EDS1–PAD4 complex promotes SA biosynthesis and maintains SA‐dependent resistance mechanisms (Zhou et al. 1998), but phytoplasma effectors may modulate this defence network (Lu et al. 2014) by interfering with plant hormone pathways (Dermastia 2019; Oshima et al. 2023). The reduced FDp susceptibility of *Pad4* mutants therefore supports a regulatory role of PAD4 in defence against phytoplasma infection. In terms of possible applications in grapevine, bimolecular fluorescence complementation experiments have demonstrated that *V. vinifera* EDS1 (VvEDS1) physically interacts with both *A. thaliana* PAD4 (AtPAD4) and its *V. vinifera* orthologue, VvPAD4 (Gao et al. 2014). A recent comprehensive grapevine genome analysis identified two *AtPAD4* orthologues and six *AtEDS1* orthologues, suggesting a more complex SA‐mediated defence system in grapevine than in *A. thaliana* (Goyal et al. 2021). So, despite the challenges in predicting cascade effects on hormone balance, *PAD4* should be strongly considered in future grapevine genome‐editing studies. In *A. thaliana*, *ATL2* is a member of the ATL multigene family, encoding transmembrane RING‐H2 zinc‐finger E3 ubiquitin ligases involved in ubiquitination (Guzmán 2012). ATL2 localises to membranes and is induced by exogenous chitin, playing a central role in defence against fungal pathogens as *Alternaria brassicicola* (Kim et al. 2024). Constitutive *ATL2* expression activates SA‐JA defence pathways, highlighting its immune role (Serrano and Guzmán 2004). Unfortunately, the *Atl2* mutant seeds did not germinate despite repeated attempts, preventing further analysis. *V. vinifera* genome contains diverse ATL genes, some aligned with *A. thaliana* and *Oryza sativa* , others unique (Ariani et al. 2016). Experimental evidence has confirmed that constitutive expression of *VviATL156* enhances resistance to *Plasmopara viticola* (the causal agent of downy mildew), probably through reprogramming of the plant transcriptome (Vandelle et al. 2021). Therefore, phenotyping this mutant for FDp resistance will be an important next step. Within the same ‘Plant stress and defence’ category, differential regulation of the bactericidal permeability‐increasing gene (*BPI*) and the phloem specific membrane protein (late embryogenesis abundant, *LEA*) in *A. thaliana*–FDp systems were not confirmed in the subsequent gene expression study.

Finally, in the ‘Plant growth regulation’ category, none of the selected genes were regulated by phytoplasma infection in *A. thaliana* in gene expression studies, although some were affected by insect feeding.

## 4 Conclusions

As discussed above in the context of gene expression analysis, the reverse genetics results revealed that three out of five mutants associated with differentially expressed genes in FDp‐infected plants exhibited distinct phenotypes. *Gsl5* and *Pad4* were more resistant to FDp infection, while *Jaz7* was more susceptible. To confirm the accuracy of our reverse genetic approach in selecting mutants based on the deregulation of a specific gene in FDp‐infected plants, we tested three additional mutants. In the first one (*Ltp* mutant), gene expression remained consistent regardless of the treatment or the time of sampling. In the second ones (*Ckx5* and *Msfp* mutants), differential expression of both genes was observed at one or more sampling times, but only in plants exposed to healthy insects (H treatment). As expected, these controls showed similar infection rates to Col‐0. Finally, we completed the reverse genetic analysis by testing a double *Pad4/Pmr4* mutant carrying both *Pad4* and *Gsl5* mutations (Nishimura et al. 2003). As with the two individual mutant lines, this double mutant had a lower probability of becoming infected with FDp than the control. The few infected plants of the most tolerant mutant showed similar phytoplasma loads as the wild type. The use of FDp load as a predictor of varietal susceptibility is still debated. Although Eveillard et al. (2016) identified it as a reliable indicator, Ripamonti et al. (2021) highlighted that bacterial load alone does not adequately predict susceptibility, as other factors, including infection rate and epidemiological significance, need to be considered. Our results are more consistent with the latter interpretation, as we observed that reduced susceptibility in specific mutant lines was not necessarily associated with proportional differences in phytoplasma accumulation. The transmission of vector‐borne pathogens, such as FDp, is a complex, multistage biological process involving the pathogen itself, as well as the plant, the insect and the environment (Jones and Naidu 2019; Tate and Schulz 2022). The outcome of vector inoculation on the plant may depend on the plant's susceptibility/resistance and/or the insect's ability to feed on plant tissues (Ebert 2019; Ripamonti et al. 2022). Similar insect feeding behaviours on the double mutant and wild‐type *A. thaliana* , as determined by EPG analysis, ruled out the possibility that the plant's reduced palatability to the vector was responsible for its lower susceptibility. The observed resistance to FDp was therefore genotype‐dependent.

These results represent preliminary steps toward the identification of genetic mechanisms underlying plant resistance to phytoplasma infection. In *A. thaliana* , RNA‐seq analysis showed that FDp infection induces a much stronger transcriptional reprogramming than CYp, with a substantially higher number of differentially expressed genes, indicating a broader perturbation of host cellular processes. This difference may reflect the distinct biological behaviour of the two phytoplasmas: CYp, adapted to herbaceous hosts, is highly virulent and causes rapid symptom development, whereas FDp, primarily associated with woody hosts such as grapevine, appears to elicit a more extensive defence response in *A. thaliana* (Trivellone and Dietrich 2021). The higher number of deregulated genes likely reflects a broader host response rather than greater FDp pathogenicity, involving interconnected defence pathways related to hormone signalling, development and stress responses. In particular, the involvement of hormone‐related pathways, such as the JA pathway previously associated with FDp resistance (Corio‐Costet et al. 2025) and recovery (Pacifico et al. 2019; Casarin et al. 2023) in grapevine, highlights the importance of validating these preliminary findings in *V. vinifera* to confirm the role of candidate genes in the natural host of FDp.

## 5 Experimental Procedures

### 5.1 Phytoplasma Transmission to *A. thaliana*

#### 5.1.1 Phytoplasmas, Plants and Insects

Flavescence dorée phytoplasma (16SrV‐C phylogenetic group, FD‐C, Arnaud et al. 2007) was transmitted initially to broad bean ( *Vicia faba* ) by the natural vector *Scaphoideus titanus* from a naturally infected grapevine ( *V. vinifera* ) of a north‐western Italian vineyard, and maintained in controlled conditions as previously described (Galetto et al. 2014), was used to infect *A. thaliana* plants using the laboratory vector *E. variegatus* as described by Rashidi et al. (2015). For RNA‐seq, we also used the Chrysanthemum yellows phytoplasma (CYp) isolate of ‘*Ca*. Phytoplasma asteris’, to inoculate *A. thaliana* Col‐0 plants using infective *Macrosteles quadripunctulatus* as described by (Pacifico et al. 2015).

Wild‐type *A. thaliana* Col‐0 and mutants used in this study (Table S6) were purchased from The Nottingham Arabidopsis Stock Centre (NASC, UK). Plants were seeded and grown in pots with soil for cactaceae (VigorPlant) and perlite (ratio 2:1) under controlled conditions on a 10‐h light and 14‐h dark cycle at 23°C.

Healthy colonies of *E. variegatus* and *M. quadripunctulatus*, originally collected in Piedmont, Italy, were routinely maintained under laboratory conditions. The insects were continuously reared on oats ( *Avena sativa* ) and kept in cages in growth chambers at a temperature of 20°C–25°C, under a 16‐h light:8‐h dark photoperiod.

#### 5.1.2 FDp Inoculation for the Gene Expression Studies

Nine pots with four wild‐type *A. thaliana* plants each (36 plants) were caged together with 40 FDp‐infective insects for a 7‐day IAP. Two negative controls were performed: *A. thaliana* exposed to healthy *E. variegatus* (K), and healthy plants not exposed to insects (H). At the end of the IAP, insects were removed and batches of 12 plants were singly sampled at 1, 3 and 4 wpi. At each date, sampled leaves were stored at −80°C until nucleic acid extraction. To exclude the potential effect of sampling stress on gene expression in subsequent analyses, plants were sampled only once.

#### 5.1.3 Phenotyping for FDp Susceptibility of *A. thaliana* Mutants

For each genotype, 30 singly potted plants were caged together with 32 FDp‐infective insects for a 7‐day IAP. At the end of the IAP, insects were removed. Plants were sampled at 4 wpi for FDp detection and quantification. Due to logistical restraints, a maximum of four genotypes were analysed simultaneously, together with the reference Col‐0 wild type (Table S7). The experiment was repeated once for all the selected mutants and twice for those showing different FDp susceptibilities compared to Col‐0. For all inoculation experiments, groups of insects with estimated average infectivity ≥ 80.0% were used.

### 5.2 Phytoplasma Detection and Quantification

Total DNA was extracted from 50–100 mg of plant material or from a single insect, using a modified cetyltrimethyl ammonium bromide (CTAB) procedure originally described by Daire et al. (1997). The presence of CYp and FDp was verified by quantitative PCR (qPCR) in a CFX Connect Real‐Time PCR Detection System (Bio‐Rad), according to Galetto et al. (2005) and Pelletier et al. (2009), respectively. Universal phytoplasma primers CYS2Fw/Rv and FAM‐labelled TaqMan CYS2Probe designed on ribosomal 16S rRNA gene sequence (Marzachí and Bosco 2005) were used to detect CYp. FDp was detected with the specific primers Map F/R designed on MAP gene sequence (Pelletier et al. 2009). For each sample, 1 μL of diluted DNA (30 ng/μL) was used as template in a 10 μL reaction volume. Both assays were performed using the same thermal cycling conditions: 95°C for 3 min, followed by 40 cycles of 95°C for 10 s and 60°C for 30 s. CYp detection was carried out using 1× iTaq Universal Probes Supermix (Bio‐Rad), with 300 nM of each primer and 200 nM of TaqMan probe. FDp detection was performed using 1× iTaq Universal SYBR Green Supermix (Bio‐Rad) with 300 nM of each primer, followed by a melting curve analysis to confirm amplification specificity. Each sample was run in triplicate. To quantify the pathogen load, expressed in genome units (GU) of phytoplasma per nanogram of total DNA, four 100‐fold serial dilutions of the pGEM‐T Easy (Promega) plasmid harbouring the target phytoplasma genes were included on each qPCR plate. The dilution range for all plasmid standard curves was 10^8^ to 10^2^ target copies per μL.

### 5.3 RNA‐Seq

For this experiment, healthy and phytoplasma‐infected wild type *A. thaliana* plants were sampled at symptom appearance (CYp: 18 days post‐infection, dpi; FDp: 10 wpi). A total of 6 μg of RNA, extracted from phytoplasma‐infected and corresponding control plants, was sent to Macrogen Inc. (South Korea) for ribosomal RNA (rRNA) depletion using the Ribo‐Zero Gold Kit (Epicentre), cDNA library construction with the TruSeq Stranded Total RNA Sample Prep Kit (Illumina), and sequencing on the Illumina HiSeq 2000 platform. Eight cDNA libraries (two biological replicates per condition) were generated. Adapter trimming was performed using Trimmomatic, and read quality was assessed with FastQC. High‐quality reads were subsequently mapped to the *A. thaliana* transcriptome obtained from The Arabidopsis Information Resource (TAIR, https://www.arabidopsis.org/). Differential gene expression analysis was conducted using the DESeq2 package (v. 1.14.1), applying an adjusted *p*‐value threshold of ≤ 0.01 and log_2_ fold‐change (log_2_FC) cut‐offs of ≥ 1.5 for upregulated genes and ≤ −1.5 for downregulated genes.

All RNA‐seq libraries have been submitted to the NCBI Sequence Read Archive (SRA) under BioProject PRJNA1377869.

### 5.4 Gene Expression

The regulation of the selected genes was validated in *A. thaliana* following FDp infection by reverse transcription (RT)‐qPCR as detailed by Rossi et al. (2018). Total RNA from 50 to 100 mg of plant material was extracted using TRIzol reagent (Invitrogen) following the manufacturer's instructions. For each sampling date, cDNA was synthesised from total RNA (500 ng) using a High‐Capacity cDNA reverse transcription kit (Applied Biosystems) according to the manufacturer's instructions, diluted 1:5 in double‐distilled water, and stored at −80°C until the qPCRs were performed.

All primer pairs used for qPCR are listed in Table S3. Cycling conditions were: 95°C for 3 min, and 40 cycles at 95°C for 15 s and 60°C for 30 s of the annealing/extension step. The specificity of the PCR products was verified by melting curve analysis for all samples. No‐template controls were always included in each plate. The stability of five different reference genes was validated using the geNorm software (Vandesompele et al. 2002).

### 5.5 EPG Experiments and Data Analysis

We used the EPG technique to analyse the feeding behaviours of *E. variegatus* on *A. thaliana* Col‐0 (control) and *Pad4*/*Pmr4* (mutant) plants, being the least susceptible mutants. In the absence of a specific EPG waveform characterisation for *E. variegatus* on *A. thaliana* , interpretation was based on published studies of phylogenetically and functionally related leafhopper species, notably *S. titanus* feeding on *V. vinifera* (Chuche et al. 2017; Ripamonti et al. 2022; Maluta et al. 2023; Roddee et al. 2023). EPG experiments were conducted on *E. variegatus* adults allowed to feed on the selected *A. thaliana* genotypes as described by Ripamonti et al. (2022), with a Giga‐8dd DC‐EPG amplifier (EPG Systems). Input resistance used was 1 GΩ, output set at 75× gain and plant voltage adjusted so that the EPG signal fitted into +5 V and −5 V recording window. All recordings were started between 11:00 and 11:30 a.m., ending between 7:00 and 7:30 p.m. A total of 63 recordings were done, each day a total of 8 recordings were run. Each recording was represented by a different *A. thaliana* genotype/male or female insect combination. Plants were randomly arranged in the Faraday cage for every recording and discarded after use. In case of falling from the leaf, the insect was repositioned. At the end of the recording, dead insects were excluded from further analyses. About 30 recordings were acquired for each analysed genotype (*Pad4/Pmr4* and Col‐0) and marked using Stylet + software (v. 01.30, Electrical Penetration Graph Data Acquisition and Analysis, EPG Systems). Waveform marking was conducted according to Ripamonti et al. (2022).

### 5.6 Statistical Analysis

#### 5.6.1 Gene Expression

Time course changes in relative gene expression in *A. thaliana* following different experimental treatments (H, K, FD) were analysed using the statistical software R v. 4.0.3 (R Core Team 2020). Given the high variability in relative gene expression among mutants, separate two‐way ANOVAs (Gaussian GLM) were performed for each gene, testing the effect of vector exposition treatment (three levels: no insects, healthy insects, FDp‐infected insects), time post‐inoculation (1, 3 and 4 wpi), and their interaction. The normal distribution of the data was checked with the Shapiro–Wilk normality test. Holm–Sidak test was carried out as post hoc test for multiple comparisons among different treatments and times. We then tested if the relative expression varied significantly among *A. thaliana* genes depending on vector exposition treatment and time post‐inoculation using multivariate RDA (function *rda* in R package *vegan*). RDA is a constrained extension of principal component analysis (PCA) in which canonical axes—built from linear combinations of response variables (i.e., gene expressions) – must also be linear combinations of the explanatory variables (i.e., treatment and time), fitted by multiple linear regression (MLR) (Borcard et al. 2018). Monte Carlo permutation procedures were carried out for significance testing of the gene/mutant and time variables, using *anova.cca* function in R package *vegan*.

#### 5.6.2 Evaluation of *A. thaliana* Mutant Susceptibility to FDp

The probability (*p*) of an infected insect to successfully inoculate FDp to a recipient plant during a 7‐day IAP was calculated by applying the following modified Swallow *p* formula (Swallow 1985; Bodino et al. 2023):

$$p=1-{\left(1-\alpha \right)}^{1/\left(\mathrm{\beta k}\right)}$$

where *p* is the efficiency of inoculation or probability of disease transmission by a single infected vector, *α* is the proportion of infected plants, *β* is the proportion of infectious insects, and *k* is the number of individuals used per tested plant. Hence, we obtained an estimate of the average and confidence intervals (CI) of *p* (i.e., probability of infection by a single FDp‐infected insect vector) for each *Arabidopsis* line across different trials using non‐linear least‐squares regression (nls function in package *stats*). Pairwise comparisons among *p* of different mutants were done using custom R functions assuming normal distribution of the standard error.

#### 5.6.3 EPG Analyses

EPG marked recordings (file. ANA) were transformed using the rwaves function in the package Rwaves on R software v. 4.0.3 (Ripamonti et al. 2022; R Core Team 2020). Both descriptive statistics (packages dplyr, tidyr: Wickham et al. 2019) and univariate analyses using GLMs of different distribution families specific for the nature of the dependent variable (packages stats, MASS, glmmTMB: Venables and Ripley 2002; Brooks et al. 2017) were performed with gene mutant and insect sex as independent variables for each feeding variable considered.

## Author Contributions

**Marika Rossi:** investigation, writing – review and editing, formal analysis, methodology, validation. **Luciana Galetto:** writing – review and editing, investigation, methodology, validation. **Simona Abbà:** writing – review and editing, methodology, investigation, data curation. **Nicola Bodino:** formal analysis, writing – review and editing. **Cristina Marzachì:** conceptualization, writing – original draft, funding acquisition, resources. **Sabrina Palmano:** writing – original draft, conceptualization, investigation, methodology, supervision, funding acquisition.

## Funding

This work was supported by the European Union NextGenerationEU mission 4, component 2, investment 1.1 under the PRIN call 2022 (Project: Natural and BIOtechnological genetic RESistances against Flavescence dorée for vineyard sustainability—BIORES, CUP code B53D23017600006, project code 2022FW39MT), and partly by MIDI‐FD3 (Project code: DBA.AD001.690, CUP: J65E25000080007) granted by Regione Piemonte.

## Conflicts of Interest

The authors declare no conflicts of interest.

## Supporting information


**Table S1:** List of *Arabidopsis thaliana* regulated genes upon single infection with ‘*Candidatus* Phytoplasma asteris’ (Chrysanthemum yellows isolate) or Flavescence dorée phytoplasma. Deregulated genes (infected vs. healthy) are listed according to each pathosystem, together with transcript ID, fold change (log_2_), *p* value, function and protein domain.

**Table S2:** Relative expression values of the target genes related to samples collected at 1, 3 and 4 weeks post‐inoculation. FD: *Arabidopsis thaliana* exposed to infected insects; H = non treated *A. thaliana* . K = *A. thaliana* exposed to healthy insects.

**Table S3:** List of *Arabidopsis thaliana* primers designed in this study for the amplification of the selected target and reference genes. Amplified genes are grouped according to their GO function and identified by their Locus name and Gene locus. Amplicon sizes, and PCR efficiency are also reported. In bold, reference genes selected for this study.

**Table S4:** Variability of the relative gene expression for the selected genes following the three treatments (FD; K; H) at different times post‐inoculation. Comparisons in relative gene expression among treatments and times post inoculation were performed using one‐way ANOVA (one for each target gene), followed by Tukey's post hoc test with significance level set at 0.05. Plants were exposed to infected insects (FD), healthy insects (K), no insects (H).

**Table S5:** Output of permutation test on results of RDA to assess the significance of constraint variables.

**Table S6:** List of *Arabidopsis thaliana* mutants used in this study.

**Table S7:** Flavescence dorée infection rate of different *Arabidopsis thaliana* genotypes following inoculation (1 week) with infective *Euscelidius variegatus*, in the different experimental sets. Each experimental set included the wild type Col‐0 as control. Plants were sampled for the analyses at 30 days post‐infection.

**Table S8:** Non‐linear least‐squares estimates of Swallow's *p* parameters (i.e., inoculation rate of a single insect vector during a 7‐day‐long trial) for each *Arabidopsis thaliana* mutant.

**Table S9:** Median ± SE of feeding behaviour variables. Every column reports a single combination of *Arabidopsis thaliana* genotype (*Pad4/Pmr4* and Col‐0) and *Euscelidius variegatus* sex. Every row reports a specific variable, see Ripamonti et al. (2022) for a complete description of the biological meaning of variables.

**Table S10:** Output of GLM models testing influence of mutant (Gene) and sex (Sex) among selected phloem feeding behavioural variables. Separate models have been carried out for each behavioural variable.

## Data Availability

All RNA‐seq libraries have been submitted to the NCBI Sequence Read Archive (SRA) under BioProject PRJNA1377869. Other data supporting the findings of this study are either in the main document or contained in the Tables S1–, S10.
